# Commentary: Nurses at the frontline of the Tuberculosis epidemic—strengthening care in patients across health settings

**DOI:** 10.1177/17449871261446849

**Published:** 2026-06-07

**Authors:** Lwandile Tokwe

**Affiliations:** University of Cape Town, Faculty of Health Sciences Cape Town, South Africa

**Commentary to:** Nurse-led interventions in enhancing outcomes for patients with Tuberculosis: a scoping review (Yulanda et al., 2025)

Globally, in 2024, approximately 10.7 million people developed tuberculosis (TB) (World Health Organization, 2026). Although this is noted, TB is largely preventable with support from registered nurses through the administration of immunisations such as the Bacille Calmette–Guérin (BCG) vaccine at birth (Okafor et al., 2026). In adults, prevention typically involves the provision of TB preventive therapy (Chihota et al., 2025).

Patients with active TB are seen regularly by nurses who provide care and support to improve treatment outcomes. This commentary examines a scoping review by Yulanda et al. (2025) which sought to identify and describe the main components of nurse-led interventions for TB patients. The review identified six categories of nurse-led interventions. The review results are consistent with some of the nurse-led interventions for TB patients reported in our recent nursing study (Tokwe et al., 2025), with the exception of respiratory and psychological support.

## Summary of the paper

This study by Yulanda et al. (2025) aimed to identify and describe the main components of nurse-led interventions for patients with TB. A scoping review, guided by the Joanna Briggs Institute (JBI) methodology was conducted. The Population, Concept and Context (PCC) framework was guided by JBI. The search was conducted between 2 and 25 February 2025 by the authors and followed the three-stage approach recommended by JBI. A total of 11 records were included in the review. Data were synthesised by means of comprehensive descriptive synthesis. The results from the included records revealed six major domains of nurse-led interventions for patients with TB. These include health education, psychological support, respiratory exercises, TB medication guidance, dietary support and discharge instruction. The included records suggested that these nurse-led interventions were associated with enhanced TB treatment compliance, improved pulmonary function and overall quality of life and better psychological well-being among patients with TB. The findings of this review underscore the need to strengthen nursing capacity within TB programmes, particularly in the context of increasing service demands and limited resources.

## Appropriateness of the chosen review method

The authors conducted a scoping review, and this chosen review was appropriate in achieving the aim which was to identify and map existing nurse-led interventions for patients with TB, including their main components, modes of implementation and reported outcomes. Munn et al. (2018) argued that a scoping review becomes indicated when a researcher seeks to identify and map the available evidence on a particular topic of interest. Furthermore, Khalil et al. (2025) noted that scoping reviews serve as an important tool for mapping the different types of research completed to date in a specific field of study. Given this study also sought to identify knowledge gaps with regard to understanding the current nursing practices in TB care, the use of a scoping review was appropriate.

The JBI methodology underpinned this study and was appropriate as it is rigorous in nature and an evidence-based approach to evidence synthesis and uses different types of evidence to answer research questions (Joanna Briggs Institute, 2025). In this review study, this was evidenced by the inclusion of records such as quasi-experimental, randomised control trials and prospective studies.

The authors applied a clear PCC framework (Peters et al., 2020), which facilitated the inclusion of the 11 records in this study. Given the aim of identifying existing nurse-led interventions for patients with TB, the population appropriately included individuals with different forms of TB. The concept focused on nurses leading TB-related interventions, whereas the context encompassed all healthcare settings, including primary care and hospitals, without restriction.

## Critical insights

The authors’ findings make an important contribution by showing that nurse-led interventions improve health outcomes across different healthcare settings. Through this rigorous process, the findings from the included records highlight nurses as key drivers in the management of TB within their respective clinical settings, where they implement the interventions assigned to them. Furthermore, the included records highlighted that the nurse-led interventions were associated with enhanced TB medication compliance, better pulmonary function and psychological well-being and improved overall quality of life of patients with TB. Moreover, the results of the included records emphasise the crucial role of nurses in providing health education to patients with TB that goes beyond clinical settings, fostering patient self-management and long-term treatment success.

## Strengths and limitations

The use of JBI methodology facilitated the comprehensive search strategy of this review study. The use of this comprehensive search strategy enabled a broad search strategy that the authors used in all four databases. Using JBI’s PCC framework facilitated systematic mapping of different nurse-led interventions across different health settings and populations, strengthening the descriptive and exploratory aims of the review.

A notable limitation was that most of the included records provided limited descriptions of the interventions, particularly regarding timing of implementation, procedures and targeted outcomes. Furthermore, the scoping review was not critically appraised. However, it is important to note that scoping reviews guided by the JBI typically do not include critical appraisal, as their purpose is to map the available evidence rather than provide a synthesised answer to the study question (Peters et al., 2020).

## Implications for nursing practice and policy

The study’s findings have direct implications for clinical practice and policy development:

- For nursing practice and policy, nurse-led interventions such as health education, TB medication literacy and discharge instruction are feasible for implementation in resource-constrained settings, particularly in the management of TB.
- For nursing practice, the use of breathing exercises in patients with TB may also be delivered.

## Future directions

Firstly, future research should be directed on developing standardised and clearly reported nurse-led intervention models. Secondly, the developed models should be tested on a small-scale RCT for effectiveness and sustainability.

## Conclusion

This scoping review mapped six categories of nurse-led interventions for patients with TB. The findings of this review underscore the need to strengthen nursing capacity within TB programmes, particularly in the context of increasing service demands and limited resources. Overall, the using a scoping review guided by JBI methodology was appropriate and assisted in achieving the aim of the study.

I have conducted a study on the role of nurses in managing new patients living with human immunodeficiency virus (HIV) and TB in rural primary healthcare clinics in South Africa (Tokwe et al. 2025). In the study, the initial focus was on TB management prior to initiating patients on antiretroviral therapy. These review results are similar. In my study, the nurses were at the frontline of the TB programme in the nurse-led rural primary healthcare clinics. The nurses expressed their roles of health education in newly diagnosed patients. Nurses provided health education on TB medication use, including taking treatment on an empty stomach and managing side effects such as orange urine during the intensive phase. In a rural primary healthcare setting marked by food scarcity, they also advised on nutrition, including home vegetable gardening so they could have healthy diet. Nurses supported adherence counselling and reinforced continued TB medication adherence. During discharge, the nurses advised the patients to remain adherent on their HIV medication, and they initiated patients on TPT after their active TB diagnosis is excluded. These nurse-led interventions in this study also foster a patient-centred approach which has a potential to improve TB patient’s outcomes.
